# A Semi-supervised Deep Learning Method for Cervical Cell Classification

**DOI:** 10.1155/2022/4376178

**Published:** 2022-02-27

**Authors:** Siqi Zhao, Yongjun He, Jian Qin, Zixuan Wang

**Affiliations:** School of Computer Science and Technology, Harbin University of Science and Technology, Harbin 150080, China

## Abstract

Currently, the Thinprep cytologic test (TCT) is the most popular cervical cancer cytology test technique. It can detect precancerous conditions and microbial infections. However, this technique entirely relies on manual operation and doctors' naked eye observation, resulting in a heavy workload and low accuracy rate. Recently, automatic pathological diagnosis has been developed to solve this problem. Cervical cell classification is a key technology in the intelligent cervical cancer diagnosis system. Training a deep neural network-based classification model requires a large amount of data. However, cervical cell labeling requires specialized physicians and the cost of labeling is high, resulting in a lack of sufficient labeling data in this field. To address this problem, we propose a method to ensure high accuracy in cervical cell classification with a small amount of labeled data by introducing manual features and a voting mechanism to achieve data expansion in semi-supervised learning. The method consists of three main steps, using a clarity function to filter out high-quality cervical cell images, annotating a small amount of them, and balancing the training data using a voting mechanism. With a small amount of labeled data, the accuracy of the proposed method in this paper can reach to 91.94%.

## 1. Introduction

Cervical cancer is one of the most common cancers which directly threats women's lives. According to the 2018 Global Cancer Report published by the World Health Organization/International Agency [1], there were 570,000 new cases and 311,000 deaths of cervical cancer. China is a region with a high incidence of cervical cancer, about 100,000 new cases each year. These cases account for a quarter of the new cases in the world. In the last decade, the incidence and mortality rates of cervical cancer in China have been increasing, and the age of onset gradually becomes younger. The cure rate of early cervical cancer is 91.5%, so regular screening, timely detection, and treatment of precancerous conditions are effective ways to fight against cervical cancer. Currently, the Thinprep cytologic test is an effective method for screening cervical cancer. However, this method relies on manual manipulation and visual observation of the pathologists to search for abnormal cells, which brings heavy workload. At the same time, pathologists inevitably make 10-20% of misdiagnosed and missed cases due to subjectivity and visual fatigue caused by long hours of work. In addition, the accuracy of primary pathology diagnosis in China is low, and pathologists are severely insufficient. Recently, artificial intelligence and big data technologies have been used in pathology diagnosis, leading to the emergence of intelligent auxiliary diagnosis system. Capable of scanning pathology slides, intelligent diagnosis, and information management, the system can effectively improve the efficiency of pathologists and significantly reduce the operating costs of pathology departments. The core tasks of the intelligent companion diagnostic system are abnormal cell detection and pathological graded diagnosis, both of which are technically dependent on cervical cell classification. Therefore, the accuracy of cervical cell classification is an important factor in the intelligent auxiliary diagnosis system.

In cervical cell image classification, the convolutional neural network (CNN) has shown great advantages. CNN extracts local region features of images by convolutional structure, uses the same convolutional kernel in each layer of convolutional operations (parameter sharing) in order to reduce the number of parameters, and achieves displacement invariance of the output images by using pooling layers [2]. Single-layer CNNs are limited in image feature extraction. In order to improve the performance of image recognition, researchers have proposed a series of new models by adding the number of layers of convolutional and pooling layers, among which the typical ones are the AlexNet [3], VGGNet [4], GoogLeNet [5], ResNet [6], ResNeXt [7], and DenseNet [8]. Among these neural networks, the VGGNet, ResNet, and ResNeXt perform better and are more widely used in the field of medical images.

The innovation of VGGNet is to use successive stacks of 3 × 3 small convolutional kernels instead of the larger convolutional kernels in AlexNet. In fact, the structure of multiple layers of smaller convolutional kernels and smaller pooling kernels not only extracts more image features but also controls the number of model parameters and speeds up model training in the same sensory field. Deepening the network in a single way by stacking small convolutional kernel layers is prone to gradient disappearance and nonconvergence. Jaworek-Korjakowska et al. added dense layers to VGG19 and obtained 87.2% accuracy in the classification of melanoma thickness, meeting the requirements for preoperative assessment of lesion site thickness [9].

ResNet adds a constant mapping before the activation function, which solves the irreversible information loss caused by Relu (a nonlinear activation function), making itself more flexible and no longer limited by the number of layers. ResNet has a strong feature extracting capability due to the large number of convolutional layers. However, the deeper layers lead to dramatic increase in hyperparameters and require more memory and longer time for training. Lai and Chang used a modified ResNet50 to classify eight types of colorectal cancer tissue with an accuracy of 94.4% [10].

ResNeXt simplifies the ResNet network structure by incorporating a strategy of group convolution which turns single-way convolution into multiway convolution with multiple branches. With equal parameter, ResNeXt performs better than ResNet, but ResNeXt runs slower than ResNet in hardware execution. Li et al. added a spatial attention refinement module to ResNeXt-50 to calculate breast density on breast ultrasound images for early screening of breast cancer [11].

Deep learning networks are noticed by researchers because of their outstanding classification results, but they need large amounts of high-quality labeled data. The mainstream public image datasets for deep learning all have tens of thousands of images. For example, MNIST contains 10 classes with a total of 70,000 handwritten digital images; MS-COCO contains 80 classes with a total of over 200,000 labeled data; ImageNet has a total of about 1.5 million images. In contrast, public datasets related to medical images are not only few in number but also small in size, among which the public datasets for cervical cytopathological smears are Herlev and SIPaKMeD [12]. Herlev contains 917 single cell images, which divided into 7 categories based on nucleoplasm ratio and nucleoplasm brightness. SIPaKMeD contains 4049 annotated data, which divided into 5 categories based on the characteristic morphology of the cells. Current public cervical cell annotation datasets are not sufficient for the needs of deep learning networks, both in numbers and categories. In addition, the cost of annotating cervical cytopathology images is high because of the complexity of the annotation process and the high level of expertise required of the annotators. It is difficult to obtain a large amount of cervical cell annotation data by manual annotation. The VC dimension of the deep learning algorithm is redundant when annotated data is lacking, leading to overfitting and a significant decrease in classification accuracy [13].

The classification of cervical cells faces two problems in terms of data. The amount of data is extremely imbalanced in different categories. The total amount of data is sparse. Combined with deep learning networks, semi-supervised classification can reduce the impact on performance caused by of the lack of labeled data. With small amounts of labeled data, semi-supervised classification can learn good models by using large amounts of unlabeled data. Current methods fall into the following categories: self-training, multiview training, self-embedded training, collaborative training, and method based on neighbor structure. In self-training methods, a small amount of labeled data are used to train a model. The model is used to classify the unlabeled data to obtain pseudo-labeled data that are used to expand the training set [14]. In multiview training methods, different models are trained with the same data under different views. Different models can complement each other to improve the accuracy of classification. The idea of multiview methods is that the determination of category boundaries depends on the distribution of divergent samples [15]. The self-embedded methods are implemented based on the low-density separation hypothesis. The assumption is that there are significant differences between the data of different categories. In other words, the data density is low at all category boundaries. Virtual adversarial training [16] is a self-embedded method. This method enhances the robustness of the model by adding noise interference during model training. In original co-training methods, the neural network model is replicated in two copies and trained separately. It is required that the two models have the same prediction results for the same data. The mean teacher algorithm [17] is a classical co-training method. Neighbor structure-based semi-supervised classification methods rely on label propagation algorithms [18]. These methods mine hidden relationships between similar data in terms of data correlation. These hidden relationships are usually used to guide the classification. Self-training methods are widely used because they are easy to implement. However, the classification accuracy of these methods is influenced by the reliability of pseudo-labeled data and the balance of training data. In multiview training methods, multiple deep learning classifiers are to be freely combined. These methods focus on the divergence of opinion among different classifiers. In order to accomplish the cervical cell classification task, our method is based on self-training methods, incorporating the idea of multiview training and combining semi-supervised classification methods with the deep learning networks VGGNet, ResNet, and ResNeXt. In our method, different classifiers are assigned different weight, and pseudo-labels of unlabeled data are determined by classifier voting. In addition, cervical cell features are used to balance the data when filtering the samples.

To address the problems of blurred images, incorrect “pseudo-labeled,” and unbalanced data in the multiclassification task of cervical cells, three data processing steps are added to the semi-supervised method: data filtering and enhancement based on clarity evaluation, classification and filtering of unlabeled samples, and data balancing of training samples. First, in order to filter out high-quality images, the clarity of the cervical cell images is evaluated by a grey-scale variance product function, and the blurry images were deblurred by a blind image deblurring method based on regularization. Second, a small number of high-quality images are annotated as training data. VGG19, ResNet18, ResNet50, ResNeXt29_2∗64d, and ResNeXt29_4∗64d networks are trained to obtain multiple classifiers. After adding different weights to each classifier, the classifiers are combined to obtain a combined classifier. Classification results and confidence levels were obtained using a combined classifier to predict unlabeled images. Directly adding “pseudo-labels” to high-confidence images and then adding them to the training data can lead to an imbalance between the cervical cell categories and reduce the accuracy of the classifier. In order to solve this problem, the cervical cell data of different categories are processed by upsampling or downsampling, so that the number of cells in each category is equal. In sampling, the cosine similarity of every two cell images in the same class is calculated, and the cell image with the greater difference in specificity is selected. Finally, the best classification model is obtained after several rounds of expanding the data and training the model. Experimental result demonstrates that this method can effectively improve the classification accuracy of cervical cells with a small amount of annotated data and meet the practical application needs of intelligent cervical cancer diagnosis tasks.

## 2. Materials and Methods

### 2.1. Application Domain

Cytopathology is a discipline that studies the causes and pathogenesis of diseases, as well as the patterns of changes in the physiological functions of cells during the development of diseases. The most important application of cytopathology is the diagnosis of tumors, in which the presence or absence of tumor cells is determined through the examination of cytological specimens (e.g., sputum, urine, chest and abdominal fluid, and cervical smears). If cells with typical abnormal morphology are found during an the examination, it can further determine the lesion level of the tumor cells. Cervical cancer cytology screening is the most successful example of cytopathology application. This screening has significantly reduced the incidence and mortality of cervical cancer worldwide by observing cervical exfoliated cells to detect cervical cancer and precancerous conditions. Quantitative DNA cytology and TCT are often used together during cervical cancer screening, which can improve the sensitivity and specificity of early cervical cancer diagnosis. Studies have shown that DNA heteroploid cells can be found in all levels of cervical lesions. The higher the level of cervical lesion, the greater the frequency of DNA heteroploid cells. In addition, the increase in DNA content is earlier than the change in cell morphology during carcinogenesis.

The quantitative DNA ploidy analysis technique measures the DNA content in the nuclei of specially stained cervical cells by using the Lambert-Bier principle. Under normal physiological conditions, human cells have 23 pairs of chromosomes. The content of DNA in the nucleus is fairly constant. During the proliferation period, the chromosomes in the cell replicate themselves. The DNA content of normal cells is set to *N* and that of proliferating cells to 2 N. Affected by oncogenic factors, cells are genetically mutated, resulting in structural or quantitative changes in chromosomes. In addition, cancer cells are able to proliferate indefinitely. During proliferation, chromosomes continuously replicate themselves. The DNA content in the cell nucleus accumulates to 2.5 N and above. It is possible to determine whether a cell section is cancerous by measuring the relative amount of DNA or the number of proliferating cells. The Feulgen staining method is commonly used for quantitative DNA ploidy analysis techniques. There is strong adsorption between DNA and Feulgen staining molecules. The darker the nucleus staining, the higher the DNA content. However, neutrophils contain DNA and impurities can adsorb Feulgen staining molecules. These can affect the determination of DNA content in cells. Therefore, it is necessary to distinguish the nuclei of epithelial cells, neutrophil nuclei, and garbage based on morphological features by using deep learning networks. Then, the nuclei of epithelial cells are classified as normal or abnormal based on DNA content.

**Figure 1:**
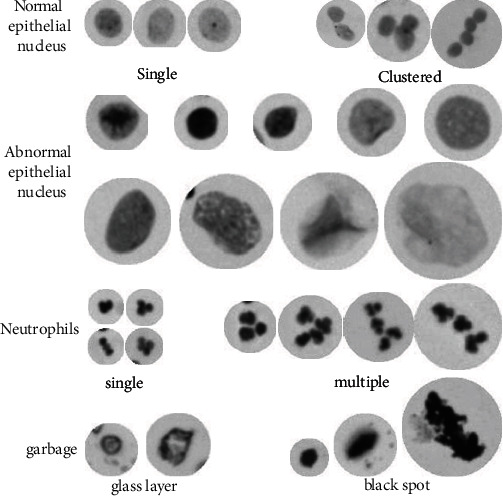
Microscopic cell diagram.

In Figure 1, neutrophils, epithelial nuclei, and garbages from different cervical samples and different periods are shown. Normal epithelial cell nuclei are round or ovoid with evenly distributed staining, and some of them contain a nucleolus (a small dark black dot in the center of the nucleus in the image). The morphology of early abnormal epithelial cell nuclei may not be altered, and only the DNA content is increased. As the time passes by, morphological changes in the nuclei of abnormal epithelial cells become apparent, such as enlarged nuclei, deviated round nuclei, and deeply stained nuclei. Neutrophil nuclei are mostly lobulated in shape, and a few are rod-shaped. The lobed nuclei are usually 2-5 lobes, with 2-3 lobes predominating in number. The garbage varies in area size and staining depth, and texture distribution is not biologically distinctive. For example, black spot garbage staining is dark and opaque, while glass layer garbage staining is light.

### 2.2. Implementation of Our Algorithm

A cervical cell classification method that combines semi-supervised learning based on self-training with classical deep learning networks is presented. The method adds several modules to the semi-supervised process, including image clarity screening, image quality enhancement, screening of pseudo-labeled data, and balancing of training samples.

#### 2.2.1. Flowchart of Our Algorithm

**Figure 2:**
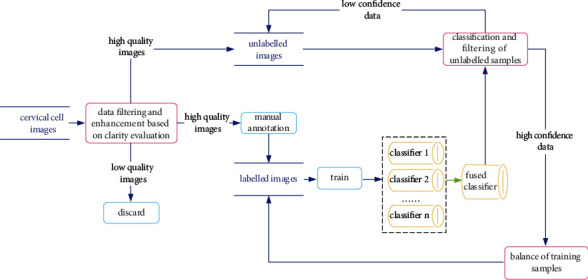
Algorithm data flow diagram.

The data flow diagram of the proposed algorithm is shown in Figure 2. The specific steps are described as follows.

First, the clarity of the cervical cell images is evaluated and high-quality images are selected. Second, a small number of high-quality images are annotated to obtain a labeled dataset, which is further divided into a training set, a validation set, and a test set. Third, deep learning networks VGGNet, ResNet, and ResNeXt are trained on the training set to obtain several classifiers. Subsequently, the classifier was evaluated on the validation set. These classifiers are then combined to obtain a combined classifier. Unlabeled images are predicted by using the combined classifier to obtain classification results and their confidence levels. Then, the unlabeled data are divided into a high confidence dataset and a low confidence dataset by comparing the predetermined trust threshold with the confidence magnitude. Next, the high confidence data set and its predicted class labels are retained, which are used to update the training sample set, and the low confidence data are put back into the unlabeled data. The training sample set is balanced. Finally, the steps of model training and training samples are repeated until the number of unlabeled data is less than 10% of the original number, and the final model is output after the end of the training round.

#### 2.2.2. Data Filtering and Enhancement Based on Clarity Evaluation

The quality of the dataset determines the performance of the classifier. Image clarity is an important indicator of the quality. To ensure the quality of the training data, the cervical cell images are screened by clarity and then enhanced.

Cervical cell images are evaluated by using grey variance product function. The grey variance product function multiplies two grayscale differences in the neighborhood of each pixel and then adds them up pixel by pixel. The basic principle is that there is a positive relationship between the clarity of a picture and the level of focus, with a focused picture containing more grey variation than an out-of-focus picture. The SMD2 function is defined as the following equation.

(1) $$\begin{array}{c} D\left(f\right)=\underset{y}{\sum }\underset{x}{\sum }\left|f\left(x,y\right)-f\left(x+1,y\right)\right|\left|f\left(x,y\right)-f\left(x,y+1\right)\right|, \end{array}$$

where *f*(*x*, *y*) is the greyscale value of any pixel in the image, and *f*(*x* + 1, *y*) and *f*(*x*, *y* + 1) are the greyscale values of the two adjacent pixels at (*x*, *y*).

After calculating the clarity mean of the images, the labeled data with clarity greater than the mean were used as the original training samples; the remaining images were processed using a regularized blind deblurring model [19] which preserved and enhanced the salient structures of images and helped to estimate the kernel functions. The model equations are described in the following equations.

(2) $$\begin{array}{c} \emptyset \left(D\right)=\int \frac{D\left(x,y\right)}{D^{2}\left(x,y\right)+\varepsilon }dxdy,\\ D\left(x,y\right)=\sqrt{u_{x}^{2}\left(x,y\right)+u_{y}^{2}\left(x,y\right)}, \end{array}$$

where *u* is an image, *u*(*x*, *y*) is the image pixel point at location (*x*, *y*), *u*_*x*_ is the partial derivative of *u*(*x*, *y*) in the *x*-direction, *u*_*y*_ is the partial derivative of *u*(*x*, *y*) in the *y*-direction, and *ε* is an arbitrary positive constant. If the clarity of the processed image reaches the average value, this image is added to the original training samples, otherwise, it is discarded.

To meet the input requirements of the deep network, the size of the cervical cell images needs to be standardized. The average edge length of the dataset images is calculated. While maintaining the aspect ratio of the original image, the length or width of the original image is randomly deflated to the average edge length, and white pixel dots are used to fill in the empty pixel areas of the image.

#### 2.2.3. Classification and Filtering of Unlabeled Samples

semi-supervised learning based on self-training for classification usually requires combining multiple classifiers to filter unlabeled images. There are two ways to combine classifiers, one is to compare the classification effects of multiple classifiers and select the best performing classifier as the recognition model for the first screening of unlabeled data; the other is to fuse multiple classifiers according to certain rules and use the fused classifier as the recognition model for the second screening.

The first filtering process is as follows: multiple models are used to predict all the unlabeled data, the classification results of each classifier for each unlabeled image and its set of confidence levels were output, and then a maximum confidence threshold was set. Different deep learning networks have different sensitivities to different classes of cervical cells when the amount of data is insufficient. Therefore, the confidence of the prediction results of different classifiers for the same unlabeled image was compared with the maximum confidence threshold, and the prediction class label of the image with confidence greater than the maximum confidence threshold was used as the pseudolabel of the data and added to the high-confidence dataset.

The secondary filtering process is as follows. The first step is to fuse multiple classifiers according to the following equation.

(3) $$\begin{array}{c} \text{Result}=\frac{\sum_{i=0}^{m}w_{i}M_{i}}{\sum_{i=0}^{m}w_{i}},M_{i}=\left[\begin{array}{c} p_{0}\\ \cdots \\ p_{n} \end{array}\right], \end{array}$$

where *w*_*i*_ denotes the weight of the *i*-th classifier, which is determined by the accuracy of the classifier in the validation set, *m* is the number of classifiers, *M*_*i*_ denotes the classification confidence vector of the *i*-th classifier, and *n* is the number of label categories. In the next step, the fusion model is used to predict all unlabeled data, outputting predicted labels, and confidence levels. Then, minimum trust threshold is set, and the confidence levels of the unlabeled data are compared to the minimum trust threshold in turn. If the confidence level is greater than the minimum threshold, the classification label corresponding to this image is treated as a pseudolabel, and this image is added to the high-confidence dataset; otherwise, the data is put back into the unlabeled dataset. In addition, if the number of unlabeled images falls below a certain proportion of the initial number of unlabeled images during the filtering process, the low confidence data are discarded directly.

#### 2.2.4. Balance of Training Samples

**Figure 3:**
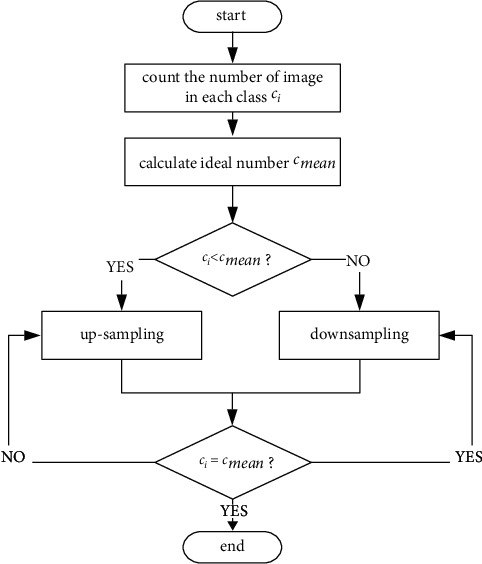
Data balancing flowchart.

To address the problem of unbalanced annotated samples of different categories in semi-supervised learning, we perform data balancing on annotated data (including original annotated data and high-confidence data) by means of upsampling and downsampling, as shown in Figure 3.

First, the label information of the annotated data (including real labels and pseudo-labels) is counted to obtain the total number of label categories *l*, the number of cells in each class *c*_*i*_ (0 ≤ *i* < *l*), the cell class with the highest number of images *c*_max_, and the cell class with the lowest number of images *c*_min_.

Second, the average of the number of images in each class (excluding *c*_max_ and *c*_min_) is calculated to obtain the ideal number *c*_mean_. The number of cell categories larger than *c*_mean_ was called rich, and those less were called rare.

Then, rich classes were randomly unduplicated downsampled several times. In the downsampling, two similar cell images were randomly selected for pairing. The cosine similarity of each pair of cell images was calculated by the following equation.

(4) $$\begin{array}{c} \cos\left(u_{i},\bar{u}\right)=\frac{\left(u_{i}-\bar{u}\right)\left(u_{j}-\bar{u}\right)}{\left\|u_{i}-\bar{u}\right\|\left\|u_{j}-\bar{u}\right\|}, \end{array}$$

where *u*_*i*_ and *u*_*j*_ are the feature vectors of the two selected cells, respectively, and $\bar{u}$ is the average of the feature vectors of all cells in the category of the two cells. The smaller the cosine angle between the two vectors, the more similar the two cells are. If the cosine angle is less than the similarity threshold, either cell is added to the training dataset and the other cell is added to the unlabeled dataset; otherwise, both cells are added to the training dataset. Integrated optical density (IOD) represents the sum of the absorbance of a certain object in the area on the picture. The calculation formula for IOD is shown in the following equation.

(5) $$\begin{array}{c} \text{IOD}=\underset{\left(i,j\right)\in \Omega }{\sum }\gamma \left(i,j\right), \end{array}$$

where *γ*(*i*, *j*) is absorbance of position (*i*, *j*). The IOD value reflects the DNA content of the cells which abnormal cells have 2.5 times more than normal cells. The integrated optical density of the nucleus is chosen as the feature vector of the cell. At the same time, to ensure that the number of images filtered out at each downsampling does not exceed half of *c*_min_, the number of downsampling *N* is determined by the following equation, where the value of *k* is chosen randomly.

(6) $$\begin{array}{c} N=\frac{c_{i}}{{kc}_{\min}},k=\frac{1}{2},\frac{1}{3}\text{or}\frac{1}{4}. \end{array}$$

Upsampling of rare classes is achieved by self-replication of real data, random rotation, and grey value transformation. Grey value transformation methods include grey stretching, inversion, logarithmic transformation, inverse logarithmic transformation, and gamma transformation. Finally, to ensure that the annotated data is balanced, the above steps are repeated before each expansion of the training set.

## 3. Results and Discussion

### 3.1. Experiments and Analysis of Results

#### 3.1.1. Introduction of Experimental Data and Groups

The public cervical cell datasets include Herlev and SIPaKMeD. Herlev is divided into 7 categories based on nucleoplasm ratio and nucleoplasm brightness. This dataset consists of 917 cell images of 128 pixels in length and width. SIPaKMeD is divided into 5 categories based on cell characteristic morphology and contains of 4049 labeled data. The staining method used by Herlev and SIPaKMeD is not suitable for DNA ploidy analysis. Public cervical cell datasets are difficult to meet the needs of deep learning networks in terms of data amount. In addition, the variety of these datasets is less. Therefore, in-house data from third-party testing centers are used in our experiments. The in-house dataset covers more categories of cervical cell images. There is greater imbalance between the data. For cell classification, this is a more difficult challenge.

**Figure 4:**
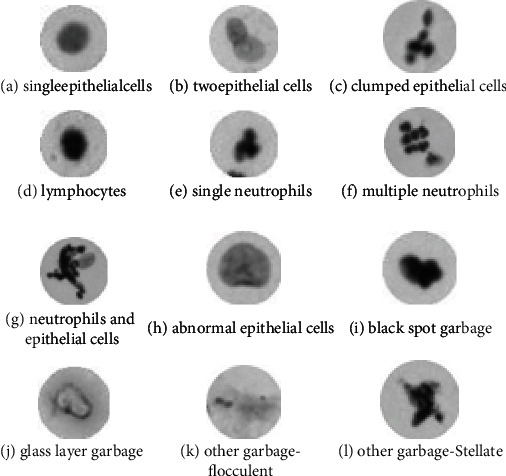
Part of typical data.

The experimental dataset was divided into 10 classes, with a total of 70,000 32 × 32 cervical cell images, 7,000 images per class. The classes are as follows: single epithelial cells (SEC), two epithelial cells (TEC), clumped epithelial cells (CEC), lymphocytes and karyopyknosis (LAK), single neutrophils (SN), multiple neutrophils (MN), neutrophils and epithelial cells (NAEC), abnormal epithelial cells (AEC), black spot garbage and glass layer garbage (BSGAGLG), and other garbage (OG). Typical images of different classes are shown in Figure 4.

The experiments were divided into two groups (A and B). Group A used supervised learning, and group B used semi-supervised learning. Group A consists of group A1 and group A2, with A1 containing 50,000 real-label data in the training set and A2 containing 10,000 real-label data. Group B consists of groups B1 and group B2, with the training set in B1 including low confidence data and that in B2 excluding low confidence data.

#### 3.1.2. Evaluation Metric, Loss Function, and Optimizer of Experiment

The evaluation metric for these experiments is the macrochecking rate (in top-1) given by the following equation.

(7) $$\begin{array}{c} P=\frac{\text{TP}}{\text{TP}+\text{FP}},\text{macro}‐P=\frac{1}{n}\overset{n}{\underset{i=1}{\sum }}P_{i}, \end{array}$$

where TP is the number of true cases, FP is the number of false-positive cases, *P* is the accuracy rate, and *n* is the number of cell categories.

The experiments used cross-entropy as a loss function with the following equation.

(8) $$\begin{array}{c} \text{loss}=-\overset{n}{\underset{i=1}{\sum }}y_{i}\log\left(y_{i\_}\right), \end{array}$$

where *y*_*i*_ is the true probability distribution of the training data of *i*-class, and *y*_*i*__ is the predicted probability distribution of the training data of *i*-class.

The experiments used the Adam [20] function as the optimizer, with the initial first-order moment estimation *β*_1_ set to 0.9, initial second-order moment estimation *β*_2_ set to 0.999, *ϵ* set to 1e‐08, and the initial learning rate set to 0.001.

#### 3.1.3. Experiments and Analysis of Results

The data set is divided into training set, validation set, and test set by a ratio of 5 : 1 : 1, and the training set is further divided into unlabeled data set and labeled data set by a ratio of 4 : 1. Each group of experiments has a 0.5 probability of performing operations such as center cropping, random angle rotation, modification of luminance, and modification of contrast during the training process to enhance model robustness. Each group of experiments uses the classical deep learning networks VGG19, ResNet18, ResNet50, ResNeXt29_2∗64d, and ResNeXt29_4∗64d to train the classifiers. It trains 200 times in total and 50 times per round. The trust threshold is set at 0.9 for the first round of training and then reduces by 0.1 for each additional round of training until 0.5.

The experimental data are shown in Table 1.

The training records of the semi-supervised experimental group are shown in Table 2.

The macrochecking rate values for several classifiers in different experimental groups are recorded in Table 3. From Table 3, the ResNeXt29_4∗64 model has the highest classification accuracy. The accuracy of the best recognition model for A1 is 0.57% higher than that for B2. The average accuracy of classifiers in group B2 was 1.004% higher than that of group B1. Comparing group A and group B, it can be seen that “pseudo-labeled” data can improve the accuracy of all types of deep learning models. Comparing group B1 and group B2, we can see that discarding the data with low confidence and avoiding the incorrectly “pseudo-labeled” data can effectively improve the recognition rate of the classifier.

**Figure 5:**
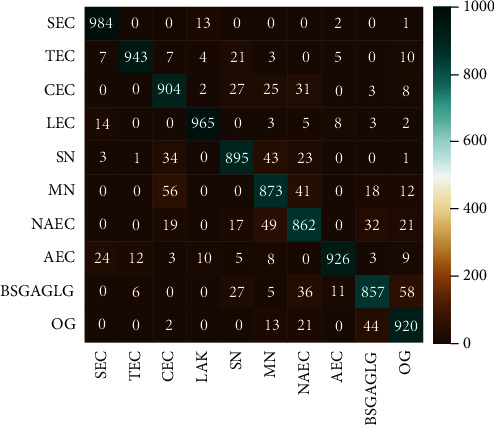
Confusion matrix of fusion classifier.

The confusion matrix of the fusion classifier on the test set in the B2 group condition is shown in Figure 5. Fusion classifier performs well in cervical multiclassification task. The classification accuracy of more than half of the classes exceeds 90%. The accuracy of the best-performing SEC class is as high as 98.4%, and the accuracy of the worst-performing BSGAGLG class also reaches 85.7%. The average accuracy rate reaches 91.9% for the cell class (the first eight classes) and 88.85% for the garbage class (the last two classes). We can see that it is easy to make error in classifying classes CEC, SN, and MN. BSGAGLG class and OG class are also easy to be classified as each other. It should be noted that the AEC samples can be misclassified into all other classes except NAEC. Affected by precancerous conditions, the AEC cells keep some features similar to those of other classes.

In Table 4, the changes in the loss values are summarized. The initial and final values are for the first and 200th training times, respectively.

**Figure 6:**
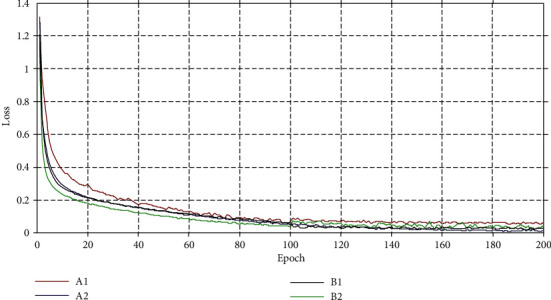
Loss value line chart.

In Figure 6, the trend of the loss curves of the fusion classifier in different experimental groups is shown. The trend of decreasing loss is obvious in the first training round, then the rate of decreasing loss becomes slower in the second training round, and the third and fourth training rounds the loss value only oscillates in a small range and gradually tends to a stable state.

We further design experiments to compare GoogLeNet, DenseNet121, FS-GCN-MIL, and SA_SVM with the proposed fusion model.

GoogLeNet introduces the Inception structure, connecting 1 × 1, 3 × 3, and 5 × 5 convolutional structures, using averaged pooling layers instead of fully connected layers, and aggregating visual features after extraction at different scales to improve model accuracy. DenseNet121 concatenates multiple Dense Blocks, with convolutional pooling between each block to maximize information flow. FS-GCN-MIL [21] uses graph convolutional networks to learn bag-level features for bag-level classification. In the method proposed in the literature [22], polynomial and radial basis function-based SA-SVM and deep network are trained using training samples randomly chosen via a bootstrap technique, and then the results are aggregated using least square estimation weighting.

**Figure 7:**
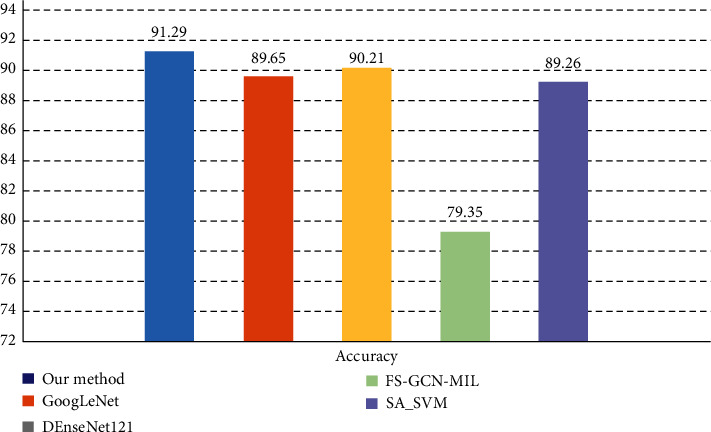
Comparison with the results of other methods.

Among them, GoogLeNet and DenseNet121 are deep learning methods, using 40,000 labeled samples for model training. FS-GCN-MIL and SA_SVM are semi-supervised learning methods, using 10,000 labeled samples and 30,000 unlabeled samples to complete the model training. The results of the comparison test are shown in Figure 7.

The experimental results show that the proposed method can effectively filter out qualified “pseudo-labeled” data from unlabeled data to train the classifier, and its accuracy is very close to that of the supervised learning group. In addition, the accuracy of this method is higher than that of the methods proposed in the literature [21, 22]. Cervical cell classification uses this paper's method instead of supervised learning to maintain high accuracy while effectively reducing the amount of annotated data.

## 4. Conclusions

To solve the problem of few data annotations for intelligent assisted diagnosis systems, a semi-supervised deep learning method for cervical cell classification is proposed. The method can accurately classify cervical cells by using a classification model trained on a small amount of labeled data and a large amount of unlabeled data. Low confidence data are difficult to identify in semi-supervised learning. If these data are used wisely, there may be a greater improvement in the recognition accuracy of the system. Our future work is to select valuable data from these data for manual annotation and to study active and semi-supervised learning with small samples. In addition, only clarity is currently selected as a reference condition in the data filtering and enhancement, and further work will consider adding other criteria, such as uniformity of illumination.

## Figures and Tables

**Figure 8:**
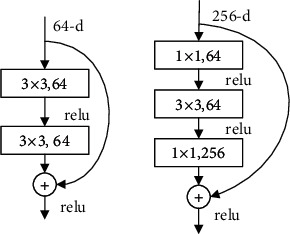
Basic units of ResNet.

**Figure 9:**
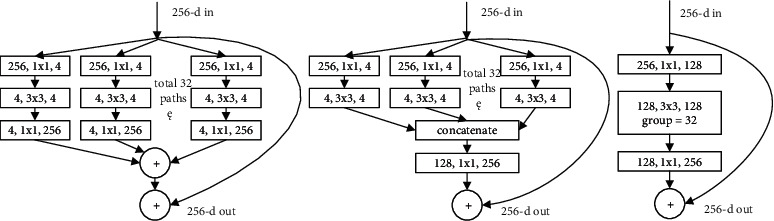
Basic units of ResNeXt.

**Table 1:** Experimental data situation of each group.

Group		The number of experimental data				
		Training set		Validation set	Test set	Discarded samples
		Real label data	Pseudo-labeled data			
Supervised learning	A1	50000	0	10000	10000	0
	A2	10000	0	10000	10000	0
semi-supervised learning	B1	10000	40000	10000	10000	0
	B2	10000	38573	10000	10000	1427

**Table 2:** Record of semi-supervised training.

Epoch	Group	Trust threshold	Labeled data	Unlabeled data	Accuracy (%)				
					Vgg19	ResNet18	ResNet 50	ResNeXt29_2∗64d	ResNeXt29_4∗64d
0	—	—	10000	40000	0	0	0	0	0
50	B1/B2	0.9	39990	10010	89.45	90.34	89.90	91.22	90.95
100	B1/B2	0.8	44140	5860	89.83	90.74	90.11	91.63	91.86
200	B1/B2	0.7	46820	3180	89.94	90.81	90.31	91.60	91.89

**Table 3:** Record of classifier accuracy.

Group	Vgg19	ResNet18	ResNet50	ResNeXt29_2∗64d	ResNeXt29_4∗64d	Fused classifier
A1	90.55%	91.60%	90.78%	93.12%	92.51%	—
A2	85.01%	87.64%	87.49%	87.21%	88.04%	—
B1	89.29%	89.44%	90.23%	90.14%	90.92%	90.32%
B2	90.14%	90.95%	90.34%	91.67%	91.94%	91.29%

**Table 4:** Record of loss value of each group.

Epoch	A1	A2	B1	B2
0	1.275	1.091	1.313	1.285
50	0.148	0.101	0.134	0.131
100	0.068	0.037	0.061	0.05
150	0.06	0.042	0.022	0.019
200	0.064	0.04	0.025	0.013

**Table 5:** Network structures of VGG.

ConvNet configuration					
A	A-LRN	B	C	D	E
11 weight layers	11 weight layers	13 weight layers	16 weight layers	16 weight layers	19 weight layers
Input (224 × 224 RGB image)					
Conv3-64	Conv3-64 LRN	Conv3-64 Conv3-64	Conv3-64 Conv3-64	Conv3-64 Conv3-64	Conv3-64 Conv3-64
Maxpool					
Conv3-128	Conv3-128	Conv3-128 Conv3-128	Conv3-128 Conv3-128	Conv3-128 Conv3-128	Conv3-128 Conv3-128
Maxpool					
Conv3-256 Conv3-256	Conv3-256 Conv3-256	Conv3-256 Conv3-256	Conv3-256 Conv3-256 Conv1-256	Conv3-256 Conv3-256 Conv3-256	Conv3-256 Conv3-256 Conv3-256 Conv3-256
Maxpool					
Conv3-512 Conv3-512	Conv3-512 Conv3-512	Conv3-512 Conv3-512	Conv3-512 Conv3-512 Conv1-512	Conv3-512 Conv3-512 Conv3-512	Conv3-512 Conv3-512 Conv3-512 Conv3-512
Maxpool					
Conv3-512 Conv3-512	Conv3-512 Conv3-512	Conv3-512 Conv3-512	Conv3-512 Conv3-512 Conv1-512	Conv3-512 Conv3-512 Conv3-512	Conv3-512 Conv3-512 Conv3-512 Conv3-512
Maxpool					
FC-4096					
FC-4096					
FC-1000					
Soft-max					

**Table 6:** Network structures of ResNet.

Layer name	Output size	18-layer	34-layer	50-layer	101-layer	152-layer
Conv1	112 × 112	7 × 7, 64, stride 2				
Conv2_x	56 × 56	3 × 3 max pool, stride 2				
		$\left[\begin{array}{c} 3\times 3,64\\ 3\times 3,64 \end{array}\right]\times 2$	$\left[\begin{array}{c} 3\times 3,64\\ 3\times 3,64 \end{array}\right]\times 3$	$\left[\begin{array}{c} 1\times 1,64\\ 3\times 3,64\\ 1\times 1,256 \end{array}\right]\times 3$	$\left[\begin{array}{c} 1\times 1,64\\ 3\times 3,64\\ 1\times 1,256 \end{array}\right]\times 3$	$\left[\begin{array}{c} 1\times 1,64\\ 3\times 3,64\\ 1\times 1,256 \end{array}\right]\times 3$
Conv3_x	28 × 28	$\left[\begin{array}{c} 3\times 3,128\\ 3\times 3,128 \end{array}\right]\times 2$	$\left[\begin{array}{c} 3\times 3,128\\ 3\times 3,128 \end{array}\right]\times 4$	$\left[\begin{array}{c} 1\times 1,128\\ 3\times 3,128\\ 1\times 1,512 \end{array}\right]\times 4$	$\left[\begin{array}{c} 1\times 1,128\\ 3\times 3,128\\ 1\times 1,512 \end{array}\right]\times 4$	$\left[\begin{array}{c} 1\times 1,128\\ 3\times 3,128\\ 1\times 1,512 \end{array}\right]\times 8$
Conv4_x	14 × 14	$\left[\begin{array}{c} 3\times 3,256\\ 3\times 3,256 \end{array}\right]\times 2$	$\left[\begin{array}{c} 3\times 3,256\\ 3\times 3,256 \end{array}\right]\times 6$	$\left[\begin{array}{c} 1\times 1,\;256\\ 3\times 3,256\\ 1\times 1,1024 \end{array}\right]\times 6$	$\left[\begin{array}{c} 1\times 1,\;256\\ 3\times 3,256\\ 1\times 1,1024 \end{array}\right]\times 23$	$\left[\begin{array}{c} 1\times 1,\;256\\ 3\times 3,256\\ 1\times 1,1024 \end{array}\right]\times 36$
Conv5_x	7 × 7	$\left[\begin{array}{c} 3\times 3,512\\ 3\times 3,512 \end{array}\right]\times 2$	$\left[\begin{array}{c} 3\times 3,512\\ 3\times 3,512 \end{array}\right]\times 3$	$\left[\begin{array}{c} 1\times 1,\;512\\ 3\times 3,512\\ 1\times 1,2048 \end{array}\right]\times 3$	$\left[\begin{array}{c} 1\times 1,\;512\\ 3\times 3,512\\ 1\times 1,2048 \end{array}\right]\times 3$	$\left[\begin{array}{c} 1\times 1,\;512\\ 3\times 3,512\\ 1\times 1,2048 \end{array}\right]\times 3$
	1 × 1	Average pool, 1000-d fc, softmax				

## Data Availability

Experimental data are not publicly available because they involve patient privacy and commercial confidentiality.

## Appendix

### A. Deep Learning Networks

The VGG framework contains six deep convolutional neural networks with different structures, which are shown in Table 5. The VGG network uses several consecutive 3 × 3 convolutional kernels instead of the larger convolutional kernels in AlexNet to better preserve image features and improve neural network results. In the same sensory field, the stacked small convolutional kernel structure allows for deeper networks, lighter network weight, and faster training. VGG19 consists of 16 convolutional layers and 3 fully connected layers, which is selected as one of the experimental networks.

ResNet is an ultra-deep network of up to 256 layers. It is modified from the VGG19 by adding residual units. That is, the introduction of constant shortcut connection channels in feature transfer allows features to skip one or more layers directly, ensuring the integrity of the information.

The idea of residual unit is to sum the inputs and outputs of the unit, which has two structures in its implementation. When the input dimension and the output dimension are equal, the inputs and outputs in the residual cell are added directly. The structure of this residual cell is shown in the left panel of Figure 8. When the input and output dimensions are different, there are two coping strategies. (1) zero-padding can be used to increase the dimensionality so that the input and output dimensions are equal. This strategy is applicable to shallow networks. (2) a 1 × 1 convolutional layer is used to connect the input and output layers and leads to an increase in the number of parameters and the amount of computation. This strategy is applicable to deep networks, and its structure is shown in the right panel of Figure 8.

ResNet is a truly deep learning network, solving the problem of gradient dissipation and gradient explosion that occurs as the network layers deepen. ResNet includes networks of different depths, as shown in Table 6. Considering the time cost, ResNet18 and ResNet50 are selected as the experimental networks.

ResNeXt proposes a strategy called grouped convolution, which achieves a balance between ordinary convolution and deeply separable convolution by controlling the number of variable bases in the group. The idea of grouped convolution originated from inception. The topology of each branch of inception is designed by hand, and it combines multiple convolutions of different sizes to obtain multiple sensory field information, improving the feature extraction capability. ResNeXt extracts the advantages of inception and ResNet. On the basis of inception, the topology of each branch is changed to the same structure containing multiple convolutions of different sizes. The residual units are replaced with blocks of the same topology stacked in parallel on ResNet. The basic unit block structure of ResNeXt is shown in Figure 9.

The ResNeXt improves accuracy effectively on the premise of reducing the parameter complexity and super parameters. ResNeXt29_2∗64d and ResNeXt29_4∗64d are selected as the experimental networks.
